# Construction and Analysis of Weighted Brain Networks from SICE for the Study of Alzheimer's Disease

**DOI:** 10.3389/fninf.2017.00019

**Published:** 2017-03-10

**Authors:** Jorge Munilla, Andrés Ortiz, Juan M. Górriz, Javier Ramírez, the Alzheimer's Disease Neuroimaging Initiative, Michael W. Weiner

**Affiliations:** Author Affiliations: UC San Francisco; University of Southern California; UC San Francisco University of Southern California Mayo Clinic, Rochester Mayo Clinic, Rochester; UC Berkeley; U Pennsylvania; USC; UC Davis; Brigham and Women's Hospital/Harvard Medical School Indiana University Washington University St. Louis University of Pennsylvania; Prevent Alzheimer's Disease 2020 (Chair) Siemens; Alzheimer's Association University of Pittsburgh Washington University St. Louis Cornell University; Albert Einstein College of Medicine of Yeshiva University; AD Drug Discovery Foundation; Acumen Pharmaceuticals; Washington University St. Louis; Northwestern University; National Institute of Mental Health; Brown University; Eli Lilly (Chair); BWH/HMS (Chair); University of Washington (Chair); Mayo Clinic, Rochester (Core PI) University of Southern California; UC San Diego; UC San Diego; UC San Diego; UC San Diego; UC San Diego; UC San Diego; UC San Diego; UC San Diego; UC San Diego; UC Davis (Core PI); UC Davis; UC San Diego; Mayo Clinic, Rochester (Core PI); Mayo Clinic, Rochester; University of London; UCLA School of Medicine; UCSF MRI; UC Davis; Mayo Clinic; Mayo Clinic; Mayo Clinic; Mayo Clinic; Mayo Clinic; Mayo Clinic; Mayo Clinic; UC Berkeley (Core PI); University of Michigan; University of Utah; Banner Alzheimer's Institute; Banner Alzheimer's Institute; University of Pittsburgh; UC Berkeley; Washington University St. Louis; Washington University St. Louis; Washington University St. Louis; Washington University St. Louis; UPenn School of Medicine; UPenn School of Medicine; UPenn School of Medicine; UPenn School of Medicine; UPenn School of Medicine; USC (Core PI); USC; USC; Indiana University; Indiana University; UC Irvine; Indiana University; Indiana University; Indiana University; Indiana University; UC San Francisco; UC San Diego; Prevent Alzheimer's Disease 2020; UC San Diego; National Institute on Aging; UC San Francisco; Brown University; National Institute of Mental Health; Cornell University; Johns Hopkins University; Richard Frank Consulting; Prevent Alzheimer's Disease 2020; National Institute on Aging; Oregon Health & Science University; University of Southern California; University of California - San Diego; University of Michigan; Mayo Clinic, Rochester; Baylor College of Medicine; Columbia University Medical Center; Washington University, St. Louis; University of Alabama - Birmingham; Mount Sinai School of Medicine; Rush University Medical Center; Wien Center; Johns Hopkins University; New York University; Duke University Medical Center; University of Pennsylvania; University of Kentucky; University of Pittsburgh; University of Rochester Medical Center; University of California, Irvine; University of Texas Southwestern Medical School; Emory University; University of Kansas, Medical Center; University of California, Los Angeles; Mayo Clinic, Jacksonville; Indiana University; Yale University School of Medicine; McGill Univ., Montreal-Jewish General Hospital; Sunnybrook Health Sciences, Ontario; U.B.C. Clinic for AD & Related Disorders; Cognitive Neurology - St. Joseph's, Ontario; Cleveland Clinic Lou Ruvo Center for Brain Health; Northwestern University; Premiere Research Inst (Palm Beach Neurology); Georgetown University Medical Center; Brigham and Women's Hospital; Stanford University; Banner Sun Health Research Institute; Boston University; Howard University; Case Western Reserve University; University of California, Davis - Sacramento; Neurological Care of CNY; Parkwood Hospital; University of Wisconsin; University of California, Irvine - BIC; Banner Alzheimer's Institute; Dent Neurologic Institute; Ohio State University; Albany Medical College; Hartford Hospital, Olin Neuropsychiatry Research Center; Dartmouth-Hitchcock Medical Center; Wake Forest University Health Sciences; Rhode Island Hospital; Butler Hospital; UC San Francisco; Medical University South Carolina; St. Joseph's Health Care; Nathan Kline Institute; University of Iowa College of Medicine; Cornell University; University of South Florida: USF Health Byrd Alzheimer's Institute; University of California, San Francisco; University of Southern California; UC San Francisco; University of Southern California; Mayo Clinic, Rochester; Brigham and Women's Hospital/ Harvard Medical School; UC Davis; Mayo Clinic, Rochester; UC Berkeley; Washington University St. Louis; Indiana University; Perelman School of Medicine, UPenn; USC; Perelman School of Medicine, University of Pennsylvania; UC San Francisco; Rehabilitation Institute of Chicago, Feinberg School of Medicine, Northwestern University; BWH/HMS (Chair); University of Washington (Chair); Core PI; Mayo Clinic, Rochester (Core PI); University of Southern California; UC San Diego; UC San Diego; UC San Diego; UC San Diego; UC San Diego; UC San Diego; UC San Diego; UC San Francisco; UC San Francisco; UC San Francisco; UC Davis (Core PI); UC San Diego; Mayo Clinic, Rochester (Core PI); Mayo Clinic, Rochester; Mayo Clinic; Mayo Clinic; Mayo Clinic; Mayo Clinic; Mayo Clinic; UC Berkeley (Core PI); University of Michigan; University of Utah; Banner Alzheimer's Institute; Banner Alzheimer's Institute; UC Berkeley; Washington University St. Louis; Washington University St. Louis; Washington University St. Louis; Perelman School of Medicine, UPenn; Perelman School of Medicine, UPenn; Perelman School of Medicine, UPenn; Perelman School of Medicine, UPenn; Perelman School of Medicine, UPenn; USC (Core PI); USC; USC; Indiana University; Indiana University; UC Irvine; Indiana University; Indiana University; Indiana University; Indiana University; UC San Francisco; Department of Defense (retired); University of Southern California; University of California, San Diego; Columbia University Medical Center; Rush University Medical Center; Wien Center; Duke University Medical Center; University of Rochester Medical Center; University of California, Irvine; Medical University South Carolina; Premiere Research Inst (Palm Beach Neurology); University of California, San Francisco; Georgetown University Medical Center; Brigham and Women's Hospital; Banner Sun Health Research Institute; Howard University; University of Wisconsin; University of Washington; Stanford University; Cornell University; ^1^Department of Communications Engineering, Universidad de MálagaMálaga, Spain; ^2^Department of Signal Theory, Networking and Communications, University of GranadaGranada, Spain

**Keywords:** SICE, ADNI, regression, computer aided-diagnosis, Alzheimer's disease

## Abstract

Alzheimer's Disease (AD) is the most common neurodegenerative disease in elderly people, and current drugs, unfortunately, do not represent yet a cure but only slow down its progression. This is explained, at least in part, because the understanding of the neurodegenerative process is still incomplete, being sometimes mistaken, particularly at the first steps of the illness, with the natural aging process. A better identification of how the functional activity deteriorates is thus crucial to develop new and more effective treatments. Sparse inverse covariance estimates (SICE) have been recently employed for deriving functional connectivity patterns from Positron Emission Tomography (PET) of brains affected by Alzheimer's Disease. SICE, unlike the traditional covariance methods, allows to analyze the interdependencies between brain regions factoring out the influence of others. To analyze the effects of the illness, connectivity patterns of brains affected by AD are compared with those obtained for control groups. These comparisons are, however, carried out for binary (undirected and unweighted) adjacency matrices with the same number of arcs. Additionally, the effect of the number of subjects employed or the validity of the regularization parameter used to compute the SICE have been not hitherto analyzed. In this paper, we delve into the construction of connectivity patterns from PET using SICE. In particular, we describe the effect that the number of subjects employed has on the results and identify, based on the reconstruction error of linear regression systems, a range of valid values for the regularization parameter. The amount of arcs is also proved as a discriminant value, and we show that it is possible to pass from unweighted (binary) to weighted adjacency matrices, where the weight of a connection corresponding to the existence of a relationship between two brain areas can be correlated to the persistence of this relationship when computed for different values of the regularization parameter and sets of subjects. Finally, network measures are computed for the connectivity patterns confirming that SICE may be particularly apt for assessing the efficiency of drugs, since it produces reliable brain connectivity models with small sample sizes, and that connectivity patterns affected by AD seem much less segregated, reducing the small-worldness.

## 1. Introduction

More than 40 million people are currently affected by Alzheimer's Disease (AD) in the world, being the most common neurodegenerative disease in elderly people. Unfortunately, there is not a cure for it yet, and drugs can only curb its progression. Moreover, the different neurophysiological changes that take place during the development of the illness have not been fully characterized, being these similar, particularly in the first stages of the illness, to those observed as a consequence of the natural aging process. As a result, new analytical tools that allow gaining insight into this neurodegenerative process are extremely important. The use of functional neuroimaging (Moradi et al., 2015) and/or structural imaging (Cuingnet et al., 2010; Westman et al., 2011; Chyzhyk et al., 2012; Liu et al., 2012; Termenon and Graña, 2012) has become increasingly popular as a non-invasive system for the analysis and diagnosis of AD. The majority of existing AD brain connectivity research is based on fMRI data (Zhang et al., 2015). Our work, however, utilizes Fludeoxyglucose Positron Emission Tomography (18-FDG-PET). PET images provide information of biological functions of the brain via glucose metabolism and have been extensively used for the study of AD. Single neurons are trying to be individually and simultaneously recorded with the Human Connectome Project, but meanwhile multi-scale approaches to the characterization of brain networks have been advocated (Breakspear and Cornelis, 2005; Honey et al., 2010) as the best alternative to understand the ecology of large-scale processes that feed back into the microscopic domain.

Functional connectivity refers to the coherence of the activities among distinct brain regions (Horwitz, 2003). The method traditionally used to infer dependencies between regions has been the covariance between activation levels in different brain areas. This, however, captures pairwise information and may not be able to effectively characterize the interaction of two brain regions working together while factoring out the influence of the rest of the regions. As a consequence, recent research has explored the use of partial correlations as mathematical tool applied to the AD study as a means to reveal conditional independence between regions given the rest as constant (Pourahmadi, 2013). Partial correlations are the off-diagonal entries of the inverse covariance matrix and thus models based on the inverse covariance, or precision matrix, allow identifying patterns associated to cerebral neurodegeneration. Taking into account the inherent sparseness of the brain network (Zalesky et al., 2010), Sparse Inverse Covariance Estimation (SICE), also known as Graphical Models or graphical LASSO (Least Absolute Shrinkage and Selection Operator), further allows controlling the number of zero entries in the IC matrix (i.e., the sparseness of the inverse covariance matrix) via a regularization parameter. This regularization parameter is typical for sparse computation, which is commonly employed in studies (Hilgetag et al., 2002) in which the number of analyzed regions is comparable to the number of patients and therefore the traditional maximum likelihood estimation (MLE) method cannot be employed.

The exploratory use of partial correlations for the AD study was already initiated by Huang et al. (2010). Undirected graphs were obtained in order to derive functional connectivity patterns. Dependencies are displayed as graphs with vertices corresponding to the regions and edges between two vertices indicating that these are not conditionally independent; i.e., an edge that connects two vertices or regions is present if and only if the activation levels of the regions are not conditionally independent. Ortiz et al. (2015) proved the use of this model in a discriminative way to classify between classes. Despite their relevance, these works fall short of analyzing the minimum number of subjects that are required to have significant results or providing valid ranges for the regularization parameter. Additionally, these works carry out comparisons for the same number of edges, making it not possible to compare the amount of connections across groups. This paper seeks to extend these studies analyzing the results for different sample sizes and values of the regularization parameters and showing evidences that comparisons between connectivity models are more meaningful when conducted for a fixed value of the regularization parameter while the amount of connections is used as a discriminant parameter across groups. Furthermore, we also prove in this paper that values estimated by SICE, when computed within the valid range of the regularization parameter, can be used to compute linear regression models and related to the strength of the connections, which shows a way to move from binary to weighted graphs. From a clinical perspective, we study the effectiveness of SICE with small sample sizes and analyze the changes in the connectivity patterns caused by AD: amount and distribution of connections, cluster-efficiency, characteristic path length and small-worldness.

With these aims, the rest of the paper is organized as follows. Section 2 provides details of the material and methods employed for the development of this paper; that is, the database used and the employed image pre-processing are described and some background on SICE is provided. Then, Section 3 analyzes and discusses the dependency of the estimated connectivity models with the number of patients used to compute SICE, determines the range of values for which the estimates of the SICE methods can be used to compute regression models and justifies the interpretation of these estimates in terms of persistence. Several characterization measurements are also provided to analyze the differences between the connectivity matrices across groups. Section 4 discusses the results, and finally, Section 5 concludes the paper.

## 2. Materials and methods

This section describes the database used in this work and the methods followed for the exploratory analysis with SICE.

### 2.1. Database

Data used in the preparation of this article were obtained from the Alzheimer's Disease Neuroimaging Initiative (ADNI) database (adni.loni.usc.edu). The ADNI was launched in 2003 by the National Institute on Aging (NIA), the National Institute of Biomedical Imaging and Bioengineering (NIBIB), the Food and Drug Administration (FDA), private pharmaceutical companies and non-profit organizations, as a $60 million, 5-year public-private partnership. The primary goal of ADNI has been to test whether serial magnetic resonance imaging (MRI), positron emission tomography (PET), other biological markers, and clinical and neuropsychological assessment can be combined to measure the progression of mild cognitive impairment (MCI) and early Alzheimer's disease (AD). Determination of sensitive and specific markers of very early AD progression is intended to aid researchers and clinicians to develop new treatments and monitor their effectiveness, as well as lessen the time and cost of clinical trials. The Principal Investigator of this initiative is Michael W. Weiner, MD, VA Medical Center and University of California - San Francisco. ADNI is the result of the efforts of many co-investigators from a broad range of academic institutions and private corporations, and subjects recruited from over 50 sites across the U.S. and Canada. The initial goal of ADNI was to recruit 800 subjects but ADNI has been followed by ADNI-GO and ADNI-2. These three protocols have recruited so far over 1,500 adults, with ages between 55 and 90, to participate in the research, consisting of cognitively normal older individuals, people with early or late MCI and people with early AD. The follow up duration of each group is specified in the protocols for ADNI-1, ADNI-2 and ADNI-GO. Subjects originally recruited for ADNI-1 and ADNI-GO had the option to be followed in ADNI-2. For up-to-date information, we refer the reader to www.adni-info.org.

Experiments conducted in this work use a subset of FDG-PET and T1-weighted MRI images for 241 subjects, consisting of 68 cognitively normal (CN) subjects, 103 MCI and 70 AD from the ADNI database (Alzheimer's Disease Neuroimaging Initiative, 2014). Demographic data (gender and age) of patients in the database and Mini Mental State Examination scores (MMSE) are summarized in Table 1. Additionally, MCI subject are further divided into 39 MCI converters (MCIc) and 64 stable MCI (MCIs); MCI converters are patients who were diagnosed as MCI but finally converted to AD in the term of 2 years, while stable MCI are those who remain MCI after this period.

**Table 1:** **Demographic data of patients in the database**.

**Diagnosis**	**Number**	**Age**	**Gender (M/F)**	**MMSE**
Normal (control)	68	75.81 ± 4.93	43/25	29.06 ± 1.08
MCI	103	76.39 ± 6.96	76/35	26.68 ± 2.16
AD	70	75.33 ± 7.17	46/24	22.84 ± 2.91

### 2.2. Image preprocessing

Voxel values in PET images represent activation or uptake levels. These images were first spatially normalized according to a PET template using SPM (Ashburner and Group, 2011), ensuring that each image voxel corresponds to the same anatomical position. Then, images were normalized in intensity in order to be able to compare them. This has been done as indicated in Illán et al. (2011), where the mean value of the 0.1% voxels with the highest intensity levels is selected as normalization value. Moreover, voxels whose activation or uptake is below 10% have been removed and considered as background, as these do not provide relevant information for classification but cause noise and computational overhead.

### 2.3. Background on SICE and sparse linear regression

Interactions between brain regions can be computed by correlation analysis but this does not factor out the contribution to the pairwise correlation due to global or third-party effects, and partial correlations should be adopted instead. Partial correlations are thus computed using the Maximum Likelihood Estimation (MLE) of the inverse covariance matrix since they correspond to the off-diagonal entries of the inverse covariance matrix. MLE, however, is not recommended when the sample size is not considerably higher than the number of variables; e.g., the number of patients is not higher than the number of regions of interest. If this is the case, sparse computation must be employed. SICE, also known as known as Gaussian graphical model or graphical LASSO (Pourahmadi, 2013) uses a regularization parameter that controls the number of zero entries.

Let **x**_**i**_ denote a *p*-dimension vector and ${\text{x}}_{1},{\text{x}}_{2},\ldots ,{\text{x}}_{\text{n}}\sim N\left(\mu ,\Sigma \right)$ be *n* samples measured at *p* selected ROIs which follow a multivariate Gaussian distribution where μ ∈ ℝ^*p*^ is the mean and Σ ∈ ℝ^*p*×*p*^ is the covariance. Then, Θ = Σ^−1^ is the inverse covariance (or precision) matrix, and the empirical covariance is:

(1) $$\begin{array}{l} S=\frac{1}{n}\overset{n}{\underset{i=1}{\sum }}\left(x_{i}-\mu \right){\left(x_{i}-\mu \right)}^{T}. \end{array}$$

It can be derived that the maximum log likelihood estimation of Θ under a multivariate Gaussian model can be obtained as follows:

(2) $$\begin{array}{l} \hat{\Theta }=\underset{\Theta ≻0}{argmax}\left(\text{log}\left(\text{det }\Theta \right)-tr\left(S\Theta \right)\right), \end{array}$$

where *tr*(*SΘ*) is the trace of (*SΘ*). If *S* is not singular, deriving with regards to Θ and setting it to zero, we would get, as expected, that the Maximum Likelihood Estimate (MLE) of the inverse covariance is $\hat{\Theta }=S^{-1}$. However, because *p* > *n* the empirical estimate of *S* becomes singular and a regularization must be applied so that a shrunken estimate of Θ can be obtained through a maximization of the penalized log likelihood function. In particular, the “entrywise" *l*_1_-norm regularization used in Huang et al. (2009) is also applied here so that the SICE method finds an estimate for the inverse covariance matrix $\hat{\Theta }$ of the brain regions by solving the following optimization:

(3) $$\begin{array}{l} \hat{\Theta }=\underset{\Theta ≻0}{argmax}\left(\text{log}\left(\text{det }\Theta \right)-tr\left(S\Theta \right)-\lambda ||\Theta |{|}_{1}\right), \end{array}$$

where ||·||_1_ denotes the sum of absolute values of all the entries in a matrix, and λ > 0 is the pre-selected regularization parameter. The larger the value of λ the more sparse are the estimates for Θ provided by SICE. Conversely, when λ is small the constraint has little effect and SICE becomes the conventional MLE.

Conditional independence between two variables (given the other variables in the multivariate Gaussian distribution) is reported by SICE, which can be used, as previously mentioned, to develop connectivity models. Two brain regions are connected if and only if they are not conditionally independent. Thus, connectivity models can be computed and analyzed for different values of sparseness and subject groups.

The coefficient β_*ij*_ measures the relationship between the *i*-th and the *j*-th features,

(4) $$\begin{array}{l} \beta_{ij}=-\frac{\Theta_{ij}}{\Theta_{ii}}, \end{array}$$

and given **x** = {*f*_1_, *f*_2_, …, *f*_*p*_} measured at the *p* selected ROIs, the *i*-th feature can be estimated as follows:

(5) $$\begin{array}{l} f_{i}=\underset{j\neq i}{\sum }\beta_{ij}f_{j}+\epsilon_{i},\text{for }i=1,\ldots ,p \end{array}$$

where ϵ_*i*_ is uncorrelated with all variables except *f*_*i*_, var(ϵ_*i*_) = 1/Θ_*ii*_ and cov(ϵ_*i*_, ϵ_*j*_) = Θ_*ij*_/(Θ_*ii*_Θ_*jj*_). In this paper the validity of these regression models when computed from the estimated $\hat{\Theta }$ rather than the real Θ is analyzed for the different subject groups.

### 2.4. The relevance of connectivity models. small-worldness

The characterization of networks of brain regions connected by anatomical or functional associations across groups can be used to reveal connectivity abnormalities. Anatomical connections typically correspond to white matter tracts between pairs of brain regions. Although the presence of anatomical connections suggests the potential for functional connections, such connections, may occur between pairs of anatomically unconnected regions. For the sake of clarity, in this paper we will keep (inherited from Huang et al., 2009) the term “functional" to refer to brain connectivity networks extracted from PET data. Note, however, that strictly speaking, such connectivity networks do not correspond to correlation in activity but measure covariation in glucose uptake between different regions, which can be further related to metabolic covariations.

Anatomical and functional connections, to be meaningful, must be defined on the same map of brain regions (Alemán-Gómez et al., 2006). We use here the 116-regions Automated Anatomical Labeling Atlas (AAL) to extract the features, and in particular, we focus on only 42 of these regions, distributed in the frontal, parietal, occipital and temporal lobes. Such regions have been selected for being considered potentially related to AD (Huang et al., 2009). Table 2 lists the names of the used regions and includes a number that will be used to index the node in the connectivity models. These regions will be the nodes or vertices of our brain networks while edges or arcs will be used to denote the presence or absence of the connections between nodes as an interpretation of the sparse inverse covariance. A non-zero partial correlation between two regions indicates that these are directly connected and it is represented by an arc. When this arc does not exist but there is a path between two regions, then these regions are indirectly connected. These connectivity graphs are also represented as adjacency matrices, with 42 rows and columns corresponding to the regions of interest and filled cells indicating the presence of arcs between the corresponding regions of those rows and columns. Since the connectivity graphs are undirected, the matrices are symmetric and the total number of filled cells is equal to twice the total number of arcs. Thus, to compute the number of arcs or connections from the connectivity matrix, we will not count for diagonal cells and reciprocity; i.e., element *ii* does not count and elements *ij* and *ji* count as just one.

**Table 2:** **Names and the corresponding indexes of the regions for connectivity modeling**.

**Frontal lobe**		**Parietal lobe**		**Occipital lobe**		**Temporal lobe**	
1	Frontal_Sup_L	13	Parietal_Sup_L	21	Occipital_Sup_L	27	Temporal_Sup_L
2	Frontal_Sup_R	14	Parietal_Sup_R	22	Occipital_Sup_R	28	Temporal_Sup_R
3	Frontal_Med_L	15	Parietal_Inf_L	23	Occipital_Mid_L	29	Temporal_Pole_Sup_L
4	Frontal_Med_R	16	Parietal_Inf_R	24	Occipital_Mid_R	30	Temporal_Pole_Sup_R
5	Frontal_Sup_Medial_L	17	Precuneus_L	25	Occipital_Inf_L	31	Temporal_Mid_L
6	Frontal_Sup_Medial_R	18	Precuneus_R	26	Occipital_Inf_R	32	Temporal_Mid_R
7	Frontal_Mid_Orb_L	19	Cingulum_Post_L			33	Temporal_Pole_Mid_L
8	Frontal_Mid_Orb_R	20	Cingulum_Post_R			34	Temporal_Pole_Mid_R
9	Rectus_L					35	Temporal_Inf_L 8301
10	Rectus_R					36	Temporal_Inf_R 8302
11	Cingulum_Ant_L					37	Fusiform_L
12	Cingulum_Ant_R					38	Fusiform_R
						39	Hippocampus_L
						40	Hippocampus_R
						41	ParaHippocampal_L
						42	ParaHippocampal_R

The degree of an individual node is equal to the number of links connected to that node, and therefore it reflects the importance of that node. Hub nodes are defined as those with the highest degrees; i.e., with the highest number of edges. A cluster is a group of nodes interconnected among them but isolated from the rest. Clustering coefficient of the network is indicative of segregation. “*Functional segregation is the ability for specialized processing to occur within densely interconnected groups of brain regions”* (Rubinov and Sporns, 2010). Conversely, functional integration is the ability to combine specialized information from distributed brain regions. Shorter paths imply stronger potential for integration, while large average path lengths correlate with low efficiencies for information transmission in the network. A well-designed network should combine an optimal balance of functional integration and segregation, that is, the presence of segregated modules connected (integrated) through links. The term small-worldness in the brain study field is thought to simultaneously reconcile the opposing demands of functional integration and segregation (Rubinov and Sporns, 2010).

## 3. Results

Throughout this section we will show that connectivity patterns computed from SICE vary depending on a number of parameters such as the number of subjects or the value of the regularization parameter. Such dependencies, however, as far as we know, had not been hitherto characterized. Thus, previous studies set a fixed value for the number of connections or arcs and carry out comparison between connectivity patterns with exactly this amount of arcs. Optimum values for the number of arcs were not provided either, implicitly assuming that they were equally valid and leaving usually the decision to use one or another value on the basis of the facility to carry out comparison; 60, 120, and 180 arcs are used in Huang et al. (2010), while 25, 50, 75, and 100 are employed in Ortiz et al. (2015). This section extends previous works on determining connectivity patterns with SICE (Huang et al., 2010; Ortiz et al., 2015) by:

- proving the discriminant power of the amount of arcs and thus the convenience that comparisons across groups are carried out for a fixed value of the regularization parameter λ rather than a fixed number of arcs,
- providing a range of regularization parameter values for valid regression models,
- analyzing the minimum number of subjects required to compute reliable connectivity models, and
- establishing a relationship between the absolute value of SICE and the persistence of the connections.

### 3.1. Discriminant power of the amount of arcs

**Figure 1:**
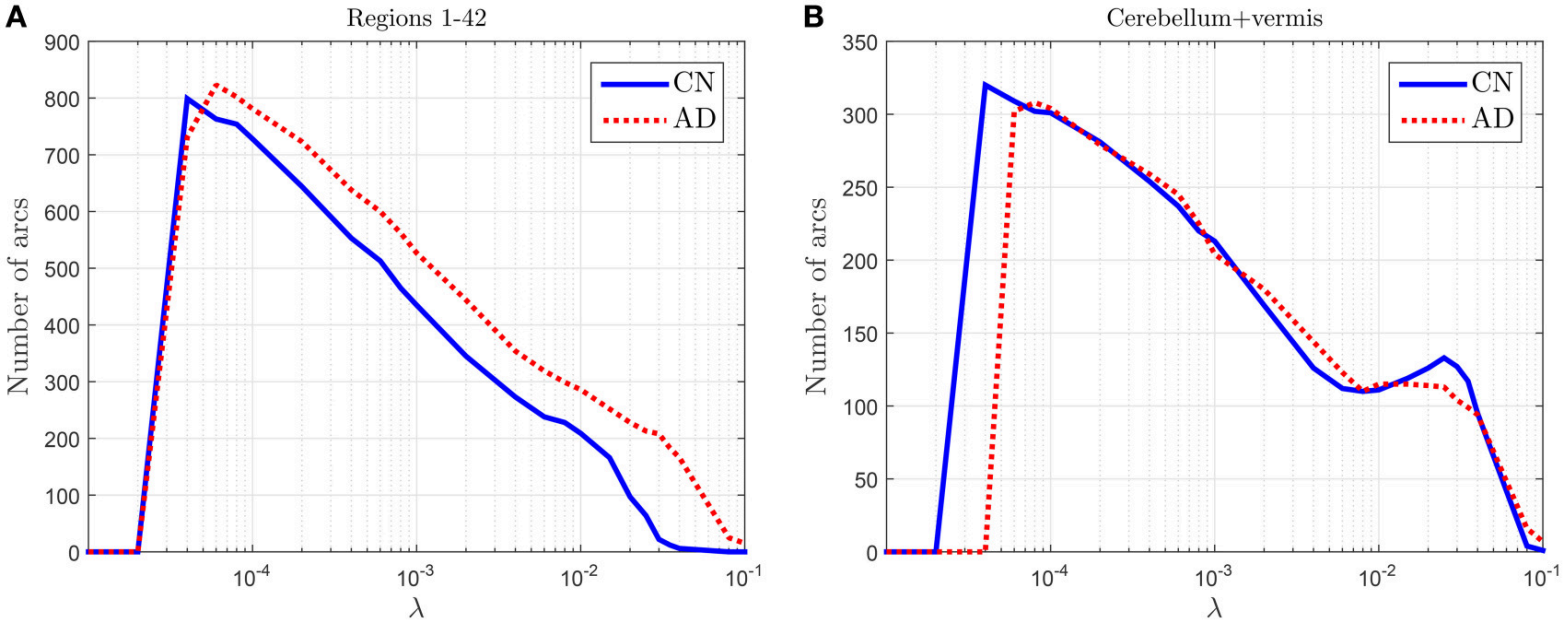
**Number of arcs for CN and AD connectivity matrices in terms of the regularization parameter for brain areas typically affected by the illness (A)** and not so affected (cerebellum+vermis) **(B)**.

To check the discriminant power of the amount of arcs an experiment is conducted where CN and AD 68-subject connectivity models are compared in terms of λ for two different cases: (i) for regions 1–42 in Table 2, which are known to be affected by AD, and (ii) for cerebellum+vermis regions, which are often used for normalization purposes since they are not or slightly affected by AD. Figure 1 shows the results of this study. The number of connections for regions not affected by the illness present similar values for both groups (Figure 1B), while it changes for areas known to be affected (Figure 1B). This result suggests that the number of connections is also a feature that can be used to characterize the effects of AD and therefore model comparisons across groups should take it into account. Naturally, when comparing models for a fixed number of arcs this information is lost. In particular, we note that the amount of connections is, in those areas affected by the illness, higher for AD patients than for CN subjects. Despite AD is usually described as a disconnection syndrome, this result is consistent with the literature, where this characterization is refined by the observation that direct links are replaced by a proliferation of alternative, indirect pathways in an attempt to maintain information transfer (Suckling et al., 2015).

This analysis also helps us to determine a coarse range, which will be fine-tuned in the next section, for the values of the regularization parameter: 4 · 10^−5^ < λ < 10^−1^.

### 3.2. Range of λ

The validity of a specific value of λ can be defined in terms of the validity of the connectivity pattern computed for such value of λ. The validity of a connectivity pattern is further defined here on the basis of the reconstruction capabilities of the regression model constructed from this connectivity pattern. Thus, in this section, we first show that values estimated with SICE, when computed within a certain range of the regularization parameter, can be used in the computation of regression models, and then, once this is proved, we determine this range of λ.

**Figure 2:**
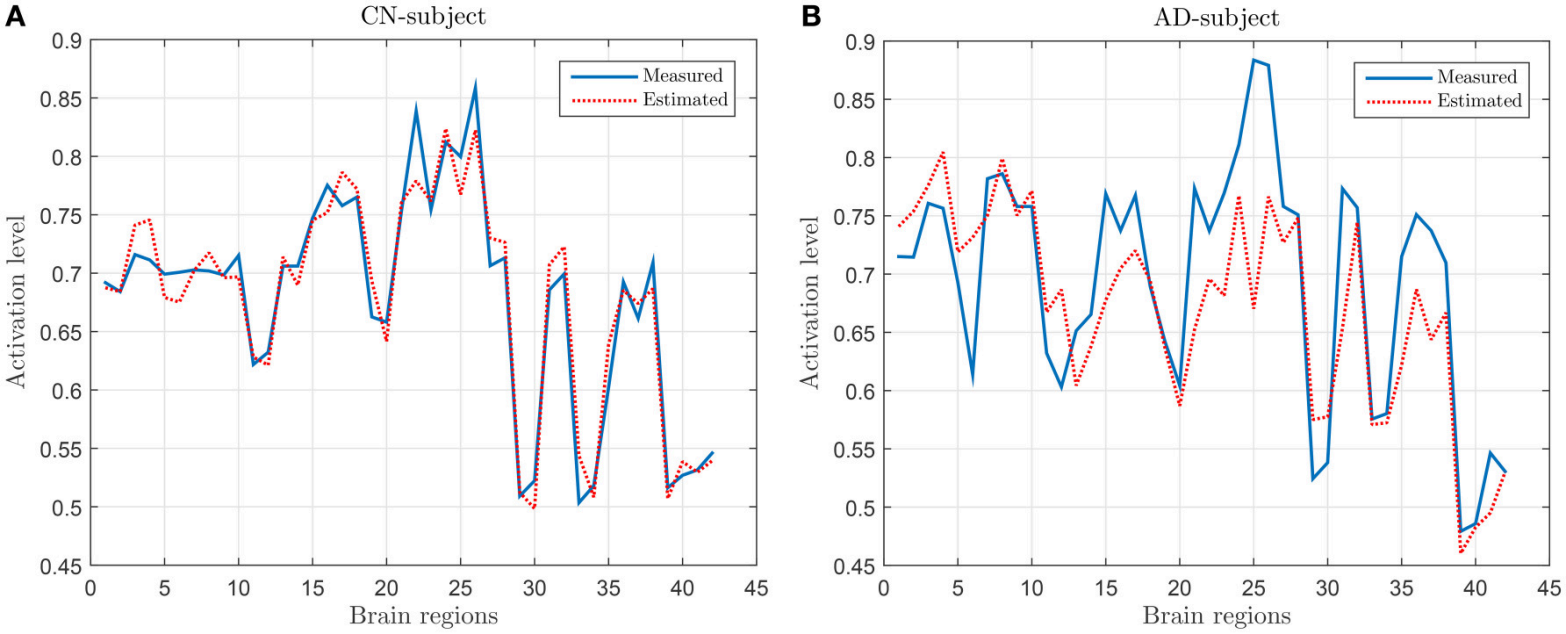
**Examples of measured and estimated values for a CN subject (A)** and AD patient **(B)**.

Following the process described in Section 2.3, a regression model has been derived by using the values of SICE computed with CN subjects at λ = 10^−2^. Figure 2 shows the measured and the estimated values, for a CN (Figure 2A) subject (who has not been used for computing SICE and the corresponding regression model) and an AD subject (Figure 2B). It can be observed that the regression model fits reasonably well the measured values and that, as expected, does better with the CN than with the AD subject.

**Figure 3:**
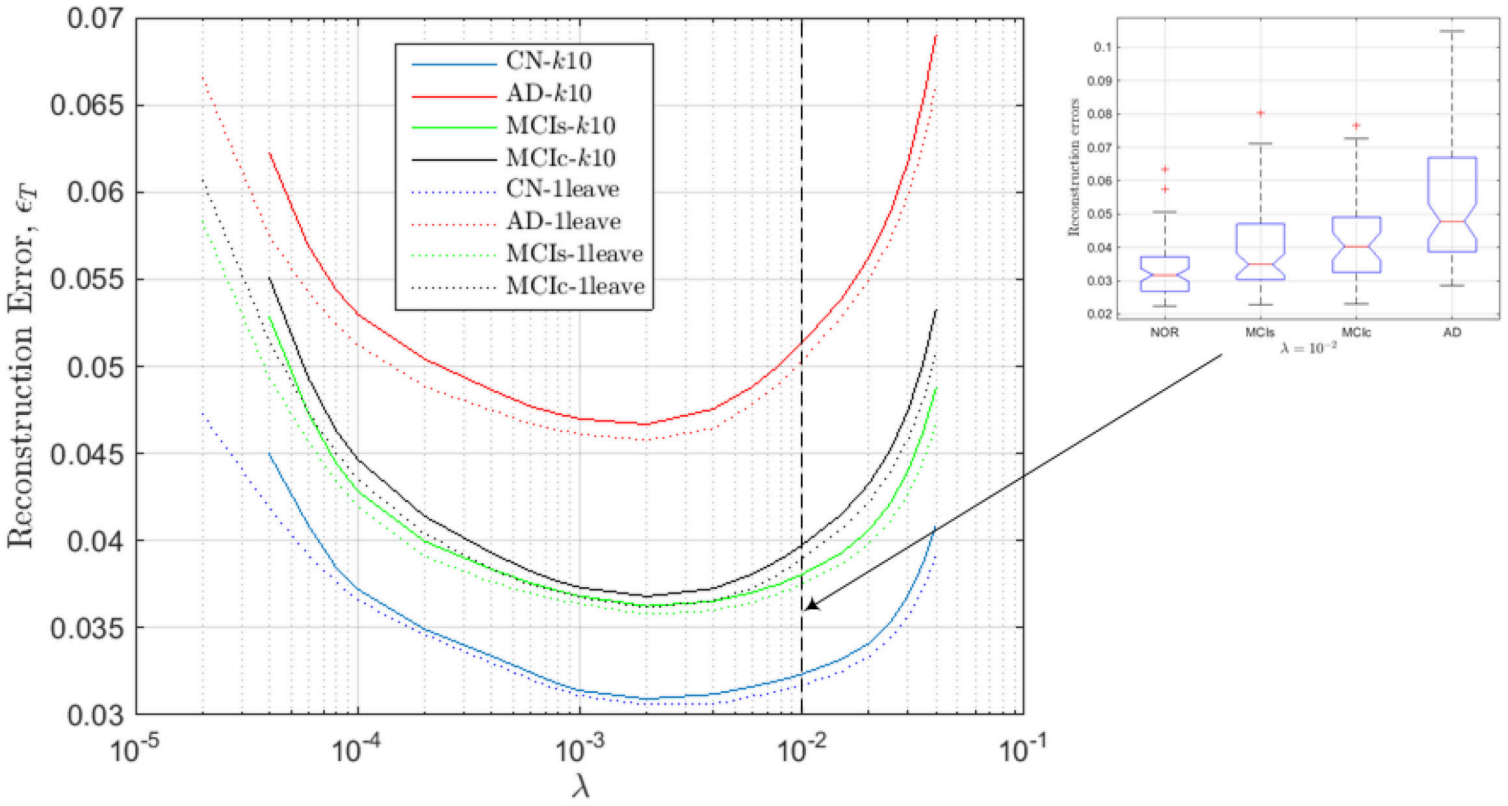
**Reconstruction errors for the regression model computed from the estimated values of SICE for the CN subjects**.

For computing the range, we now repeat this process for different values of λ and plot in Figure 3, for the different groups, the reconstruction error ϵ_*T*_ defined as:

(6) $$\begin{array}{l} \epsilon_{T}=\frac{\sqrt{\overset{42}{\underset{i=1}{\sum }}\epsilon_{i}^{2}}}{\sqrt{\overset{42}{\underset{i=1}{\sum }}P_{i}^{2}}} \end{array}$$

where $\epsilon_{i}^{2}$ is the mean square error computed for each region *i* and group of subjects:

(7) $$\begin{array}{l} \epsilon_{i}^{2}=\overset{n_{g}}{\underset{j=1}{\sum }}\frac{1}{n_{g}}{\left(f_{i}\left(j\right)-P_{i}\left(j\right)\right)}^{2} \end{array}$$

with *n*_*g*_ denoting the number of subject of the group (*n*_*g*_ = 68, *n*_*g*_ = 64, *n*_*g*_ = 39 and *n*_*g*_ = 70 for CN, MCIs, MCIc and AD respectively), *f*_*i*_(*j*) and *P*_*i*_(*j*) the regression estimate and the actual activation level for the region *i* of the subject *j*, and $P_{i}^{2}$ the mean square value of region *i*. This latter is further defined as follows:

(8) $$\begin{array}{l} P_{i}^{2}=\overset{n_{g}}{\underset{j=1}{\sum }}\frac{1}{n_{g}}P_{i}{\left(j\right)}^{2}. \end{array}$$

These values of ϵ_*T*_ are assessed by *k-fold* (*k* = 10) and *leave-one-out* cross validation so that the approximation errors are computed for subject which are not employed to derive the regression model; i.e., CN subjects used to compute the model are not employed to test such model. As a result, we note that for values outside the range, 2 · 10^−4^ < λ < 2 · 10^−2^, errors increase dramatically. Additionally, it can also be noted that the accuracy of the regression models seems to increase with the number of subjects used to compute the SICE, since reconstruction errors with *k-fold* cross validation are slightly higher than with *leave-one-out* cross validation, and that the lowest reconstruction errors are obtained for CN subjects, then MCIs, MCIc and finally AD patients, which is totally consistent with the evolution of the illness in the corresponding subject groups. The inset in Figure 3 shows the box-plot of the reconstruction errors at λ = 10^−2^, which gives an idea about the classification capabilities of the computed errors. In particular, they can be used to leverage the classification outcomes of the activation levels. The value λ = 10^−2^ is the highest value of the regularization parameter within the valid range so that the connectivity models computed at this value are the sparsest (simplest) ones within this range.

### 3.3. Sample size dependency

**Figure 4:**
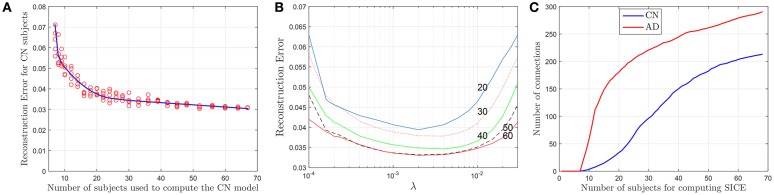
**Reconstruction errors for CN subject as a function of the number of subjects (A)**, and the regularization parameter for different sample sizes **(B)**. Number of arcs of the connectivity models as a function of the number of subjects used for the computation **(C)**.

Although SICE is known to produce reliable models with small sample sizes, the accuracy of this claim has not been assessed in previous works for this particular case. Thus, based again on the accuracy of the regression models, we characterize next the validity of these brain connectivity models. Figure 4A plots the errors for CN subjects (represented with red circles) at λ = 2 · 10^−3^ when sets (randomly selected from the sample) of different numbers of (CN) subjects are used to compute the regression model. This seems to confirm that the accuracy improves with larger sample sizes but also that SICE is able to produce reliable models with small sample sizes since the error only increases significantly when the number of subjects falls below 20. The range of valid values for the regularization parameter also remains between 2 · 10^−4^ < λ < 2 · 10^−2^ for sample sizes equal or larger than 20 (see Figure 4B). However, this increase in the accuracy has a cost in terms of complexity. There is, as shown in Figure 4C, a monotonically increasing relationship between the number of arcs in the adjacency matrices (of CN and AD) and the number of subjects (from 10 to 68) employed to compute the SICE. It must be noted, therefore, that comparisons between connectivity models for a specific value of the regularization parameter require that these are computed for the same number of subjects.

### 3.4. Weighted connectivity models

In previous works, because of the “shrinking” effect that may make that the values are not well estimated, the magnitude of the non-zero entries was disregarded and adjacency matrices were obtained by directly binarizing the IC matrix. Thus, these matrices did not provide directly any individualized information about the strength of the connections. It was defined, however, in Huang et al. (2010), an indirect quasi-measure on the basis of the monotone property, which relates the strength of a connection with the bigger value of the regularization parameter (λ) at which the connection drops. Strength was thus defined in terms of the resistance of a connection to disappear when the sparseness increases. Later, Ortiz et al. (2015) proved that the magnitude of the non-zero entries could also be directly related to this resistance of the connections to disappear when sparseness increases. Next, we check the latter and extend it to prove that the magnitudes of a SICE can also be correlated with the occurrence probabilities of their connections when computed for different sets of subjects and sample sizes.

**Figure 5:**
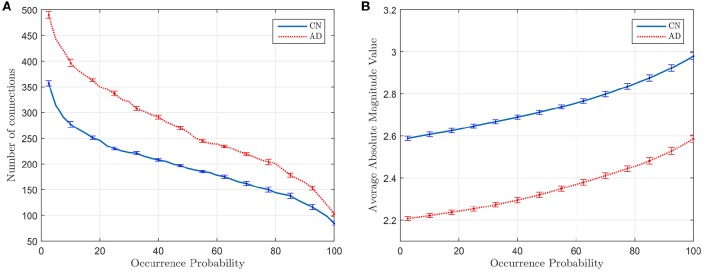
**Number of connections with equal or higher occurrence probability (A)** and mean of the magnitude value of these connections **(B)**.

Figure 5A plots the number of connections whose occurrence probability is equal or higher than a certain value when computed for 40 different random sets of 55 subjects (λ = 10^−2^), and Figure 5B represents the average magnitude value of such connections. It is clear that the latter shows a direct relationship between the average magnitude value and the occurrence probability. This probability also becomes thus a method to measure the strength of a connection. Error bars in Figure 5 indicates the standard deviation of the results of the experiment when this is repeated 20 times.

**Figure 6:**
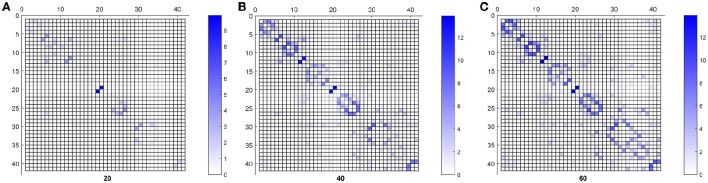
**CN weighted adjacency matrices for sample sizes of 20 (A)**, 40 **(B)**, and 60 **(C)**.

Finally, Figure 6 shows the weighted adjacency matrices computed using bootstraping as the mean of 40 different computations of SICE using groups of 20, 40, and 60 CN subjects. It serves to confirm that strength also correlates with the resistance of the connections to disappear when SICE is computed for reduced sample sizes.

### 3.5. Connectome comparisons

Once a deep insight into the use of the tool has been given, this section analyses some of its potentialities in the study of the alterations in the connectome caused by AD. In contrast to previous studies (Huang et al., 2010; Ortiz et al., 2015), the amount of connections and the strength, or occurrence probability, of these connections are included as discriminant elements.

**Figure 7:**
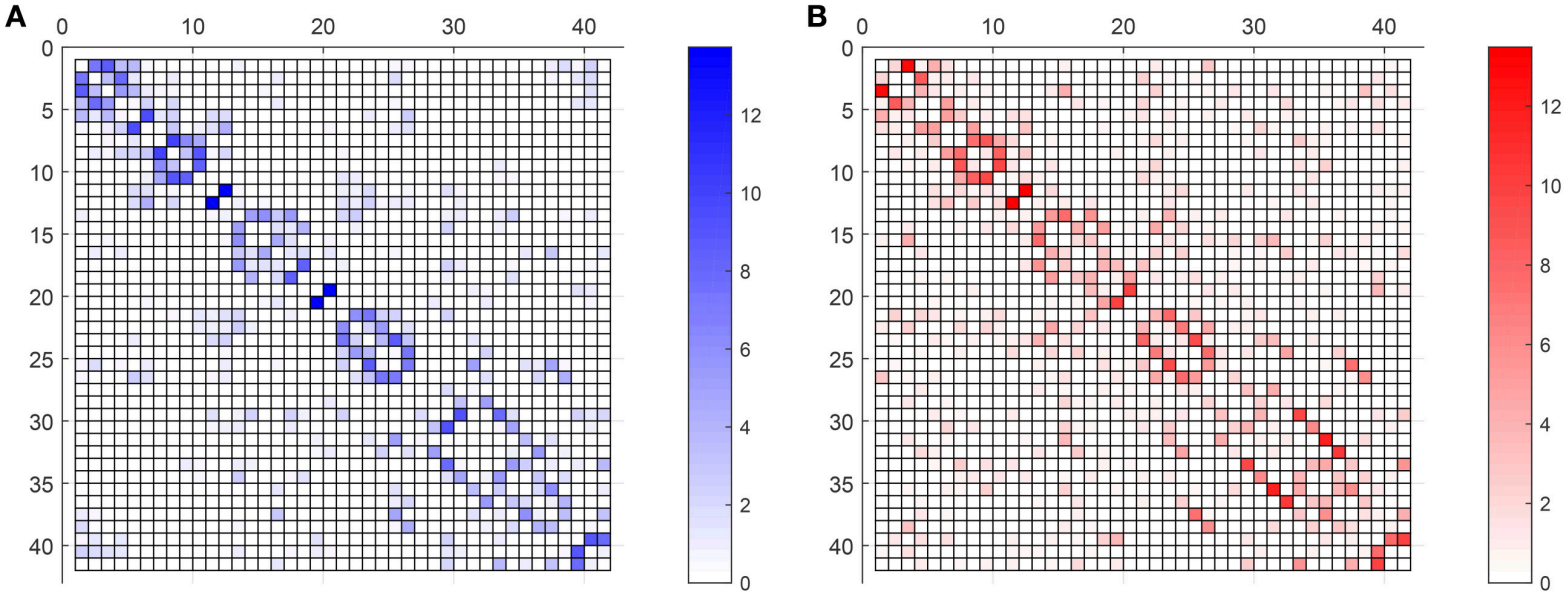
**Weighted adjacency matrices (55 subjects) for CN (A)** and AD **(B)**.

Figure 7 compares the average strength value (bootstrap for 40 different computation of SICE using groups of 55 subjects) of the connections for CN adjacency matrices and their AD counterparts. Different observations can be made: firstly, between-lobe (inter-lobe) connections are weaker than within-lobe (intra-lobe) connections. Then, the strongest connections for CN are those between the left and the right hemisphere of “Cingulum_Ant” and “Cingulum_Post.” When comparing it with the AD matrix, it is interesting to note that the strength of “Cingulum_Post” is reduced significantly, which is in accordance with the literature, which states that this area is seriously affected by AD (Grady et al., 2001; Wang et al., 2007; Supekar et al., 2008). Through the computing of the strength of the connections, we can even estimate that this strength reduction is about 27%.

**Figure 8:**
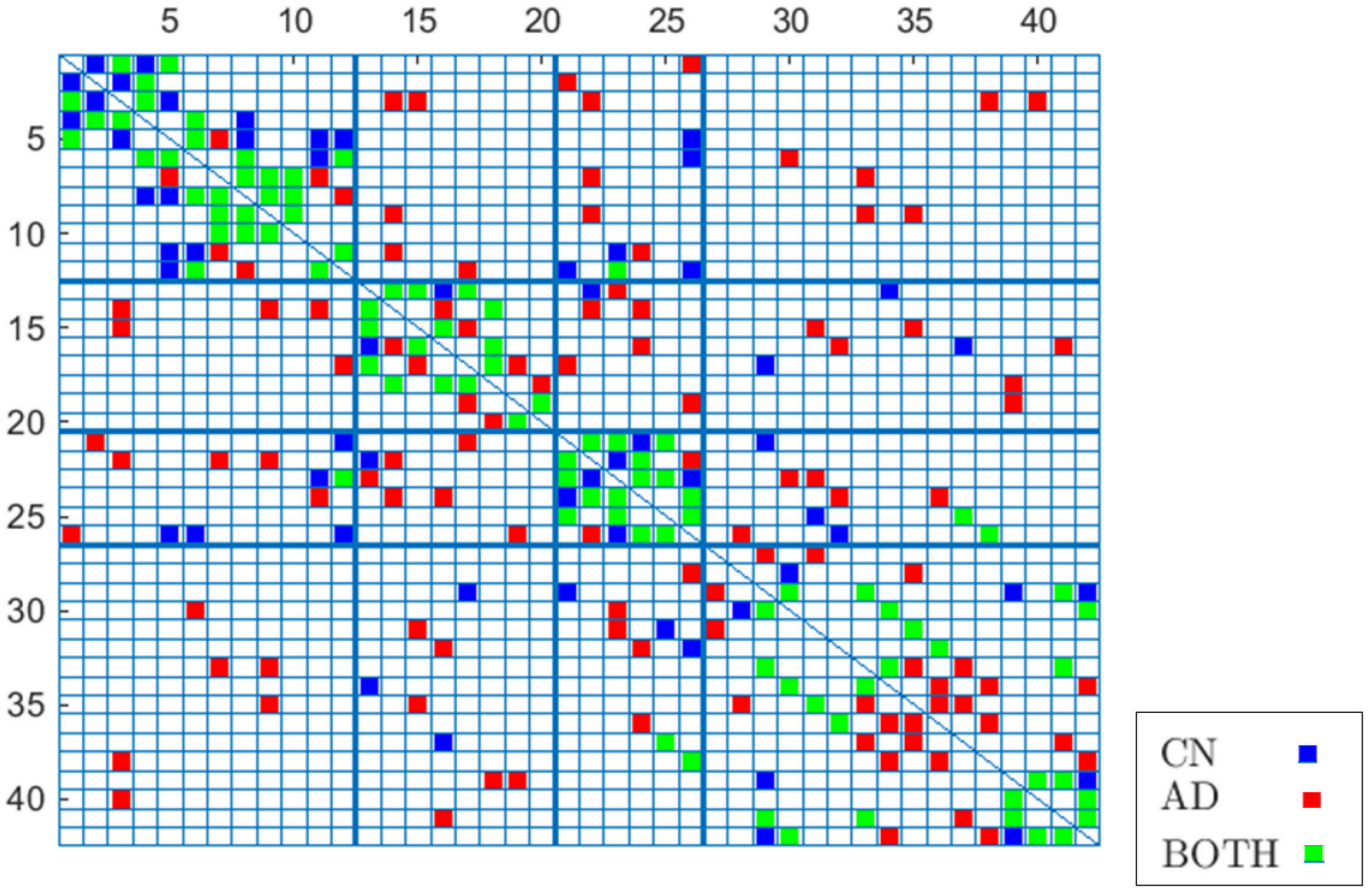
**Occurrence probability based connectome for CN and AD**.

To validate these results, Figure 8 compares again CN and AD connectomes but including only the connections that repeat in all out of 40 computations (≥ 97.5% of occurrence probability) with random groups of 55 subjects (bootstrap). Human brain tends to have a higher amount of intra-lobe than inter-lobe connections, which is clearly confirmed in the figure; the filled cells are concentrated in the diagonal of the adjacency matrix. It is also interesting to note that more than 93% of the shared links (in green in the figure) by AD and CN connectomes correspond to intra-lobe connections, while the shared inter-lobe connections represent less than 7% (all of them in the occipital lobe). For the AD connectome, in comparison with the CN, the inter-lobe connections between the frontal lobe and other regions increase, while the intra-lobe connections decrease. This is consistent with the neurology studies, which confirm that frontal lobe is typically affected in the course of the disease and explain the increase of the connectivity with other brain regions as a compensatory reallocation of recruitment of cognitive resources that could help to preserve attention ability and memory (Saykin et al., 2004; Gould et al., 2006; Huang et al., 2010; Stern, 2012).

**Figure 9:**
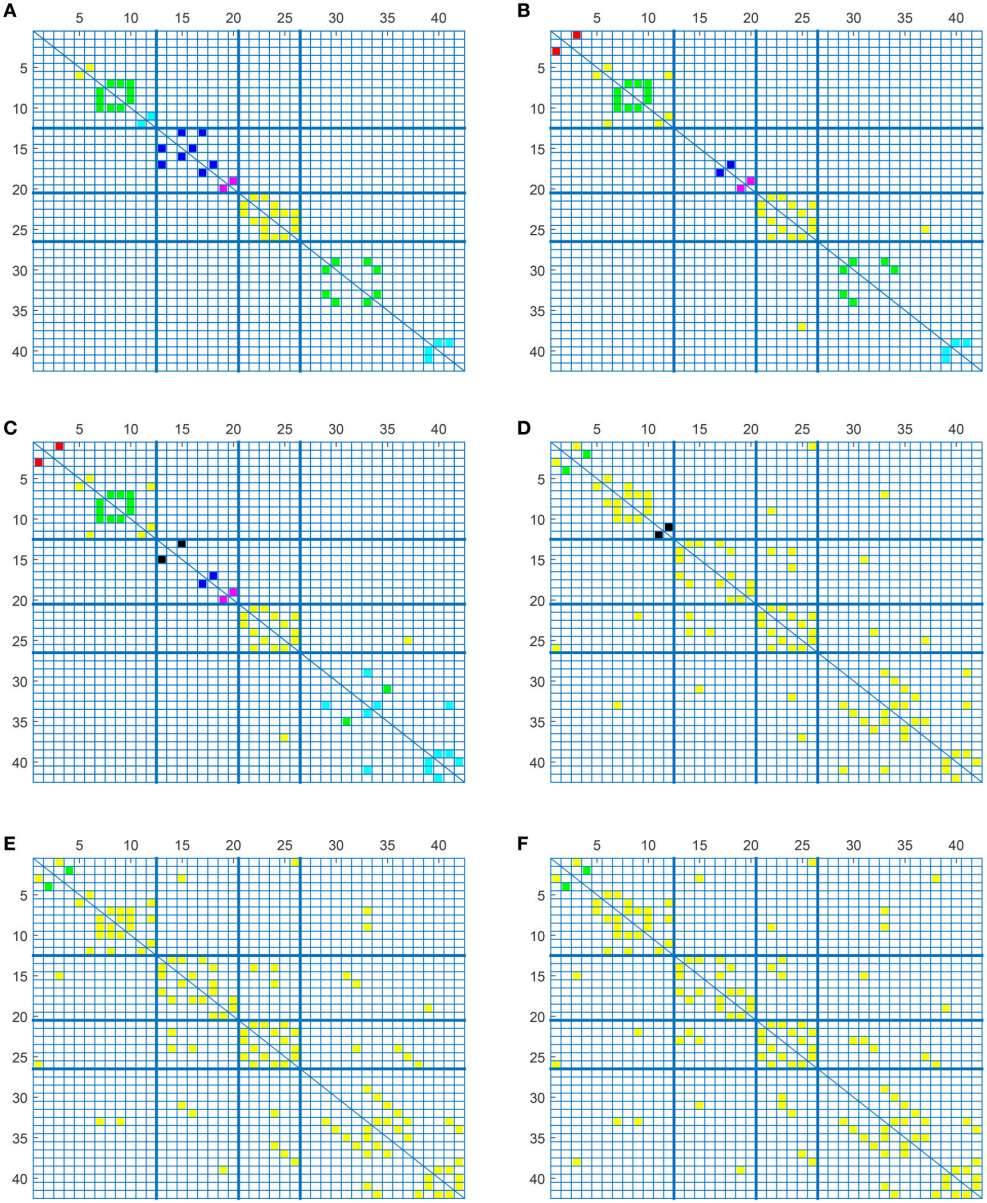
**Connectivity matrices with combined group of subjects: 40 CN (A)**, 33 CN + 7 AD **(B)**, 26 CN + 14 AD **(C)**, 14 CN + 26 AD **(D)**, 7 CN + 33 AD **(E)**, and 40 AD **(F)**.

**Figure 10:**
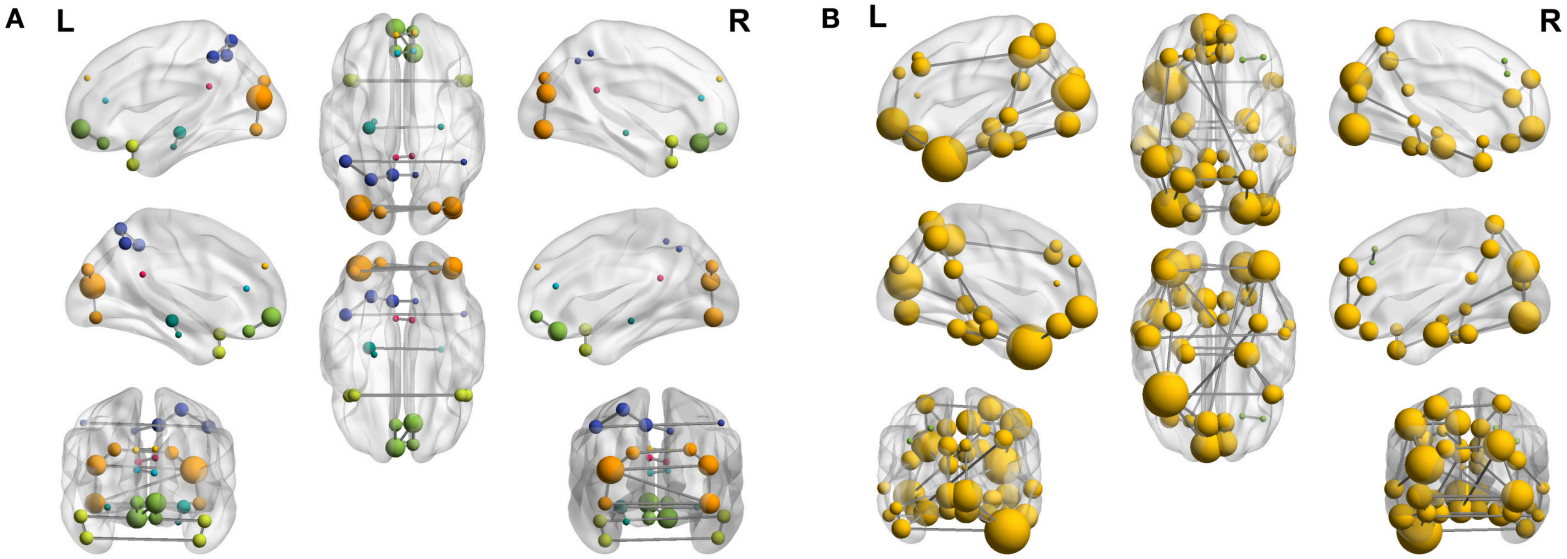
**Connectome (40 subjects) for CN (A)** and AD **(B)**.

To complete with this study, we show the evolution of the computed connectivity pattern when AD subjects are included to compute it. Thus, Figure 9 displays the evolution of the strongest connections (those that appear in all of the 40 computations) when AD subjects are included in SICE computations (for a total of 40 subjects). It is noted that the pattern for CN is initially concentrated, with very predominant intra-lobe connectivity, and gets spread when AD patients are included, as inter-lobe connectivity increases. The different clusters are plotted in different colors so that we can observe that the eight clusters that initially are present when only CN subjects are used become just 2 when only AD patients are included. At the same time the shortest path length increases, which logically should affect the small-worldness of the networks. These CN and AD brain connectivity network are compared in Figure 10 (using Brainnet Viewer Xia et al., 2013), where nodes in a cluster are represented with the same color and their sizes depend on their degrees.

**Figure 11:**
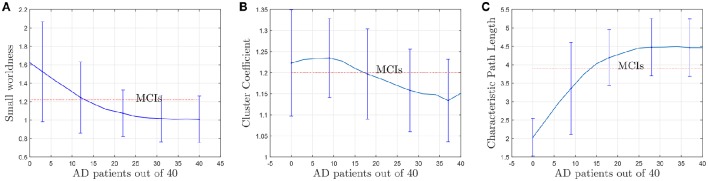
**Evolution of small-worldness (A)**, cluster coefficient **(B)** and characteristic path length **(C)** when AD patients are involved in the computation of the adjacency matrices.

As explained, small-world networks are formally defined as networks that are significantly more clustered without increasing significantly the characteristic path length. Figure 11A shows this evolution of the small-worldness in a more formal way using the rate (Rubinov and Sporns, 2010):

(9) $$\begin{array}{l} S=\frac{Clust/Clust_{random}}{L/L_{random}} \end{array}$$

where *Clust* and *Clust*_*random*_ are the clustering coefficient, and *L* and *L*_*random*_ are the characteristic path lengths of the respective tested network and a random network. Random networks are computed having a random topology but sharing the size, density and binary degree distribution of the original network (Maslov and Sneppen, 2002). Cluster coefficient (Kim et al., 2016; Zhang et al., 2016) is a measure of segregation and is the average value of the cluster coefficients of each node *C*_*i*_ computed as follows (Rubinov and Sporns, 2010):

(10) $$\begin{array}{l} C_{i}=\frac{t_{i}}{k_{i}\left(k_{i}-1\right)} \end{array}$$

where *k*_*i*_ is the degree of the node, and *t*_*i*_ is the number of triangles around the node. Since the (*i, j*) element in the *n*'th power of an adjacency matrix counts the number of path of length *n* starting at *i* and ending at *j*, the number of triangles of node *i* coincides with the value of the *i*-th diagonal element of the 3rd power of the adjacency matrix. Apart from the evolution of the small-worldness, cluster coefficients and the characteristic path lengths when AD patients are introduced for the computation are represented in Figures 11B,C. These show that the brain network evolves from a more structured small-world for CN to a less segregated network for AD. For AD, most of the nodes are connected, the averaged shortest path length is longer and the clustering coefficient becomes lower (random networks have long path lengths but also high clustering coefficients). Small-wordness, as explained, reconcile the opposing demands of integration and segregation and it is assumed to characterize brain networks (Supekar et al., 2008; Rubinov and Sporns, 2010). The values computed for stable MCI are also drawn. The results seem to confirm MCIs as an intermediate stage between CN and AD.

**Figure 12:**
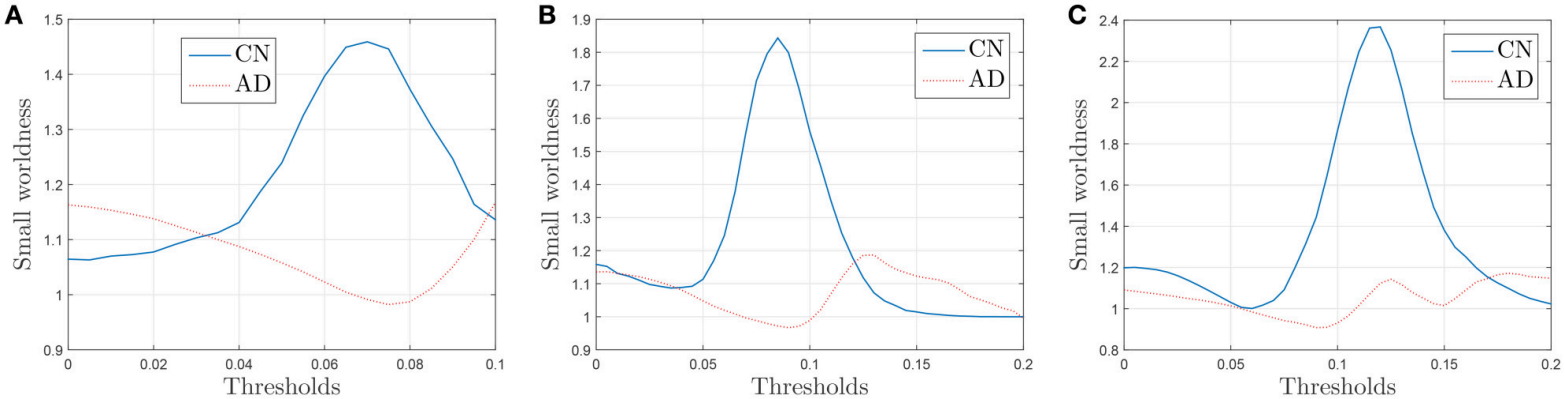
**Small-worldness against the binarization threshold for sample sizes of 30 (A)**, 40 **(B)**, and 60 **(C)**.

The results plotted in Figure 11 are the average values for 50 binarized adjacency matrices (bootstrap), each compared with the average value of 10 random matrices following the procedure described above. The threshold used for this binarization has been 10% of the maximum value (= 0.1). Not surprisingly, the exact shape of these curves is affected by the choice of this threshold (Drakesmith et al., 2015). Some authors state that this threshold must be chosen to maximize the small-worldness. Figure 12 represents the small-worldness against the threshold for different values of the sample sizes. These suggest that, to maximize the small-worldness of the CN connectivity patterns, thresholds should be gradually increased for larger sample sizes.

## 4. Discussion

This paper provides new insights into the identification of functional brain connectivity models from PET data. Using SICE we have proved that it is possible to pass from binary to weighted adjacency matrices that incorporate the strength of the connections. This strength has been also related to two different concepts from two different perspectives that have appeared to be related: (i) the probability of occurrence of a connection when computed for different group and number of subjects, and (ii) the resistance of this connection to disappear when the sparseness increases. In general, this work approaches the AD connectome problem from two different angles: (a) the use of SICE as a tool to determine functional connectivity patterns, analyzing valid ranges for input parameters, and (b) the exploratory use of such patterns in relation to the diagnosis of AD. Thus, our findings showed that:

*SICE analysis should be carried out for specified values of* λ. Previous studies based on SICE compare connectivity matrices for the same number of connections. This works, however, shows that while areas not affected by AD show similar number of connection for the same value of λ when groups of CN and AD are compared, this amount changes for areas affected by the illness. Thus, the amount of connections also becomes a discriminant parameter.

*Comparisons of the amount of connections across groups must be carried out for the same sample sizes*. The process described, based on SICE for constant values of the regularization parameter, provided us with differences in the number of connections between the groups. Specifically, AD patients show a slight reduction in the number of intra-lobe connections for the frontal and occipital lobes, but a significant increase in the number of inter-lobe connections, which can be interpreted as compensatory reallocation or recruitment of cognitive resources. By contrast, when connections between hemispheres are studied, we find that inter-hemispheric connections are slightly reduced by AD while there is an increase in the number of intra-hemispheric connections in both the right and the left hemispheres. However, we also proved that the number of arcs is affected (increased) by the number of subjects used for the computation. Therefore, comparisons across groups must be carried out for identical sample sizes, since otherwise, they may be misleading.

*SICE as a tool to compute linear regression models*. This work showed that magnitude values computed with SICE can be used to compute valid regression models provided that the regularization parameter is within a specific range; approximately 10^−4^ < λ < 10^−2^. We found that reconstruction errors are low within this range, but they increase dramatically when we move apart from it. Reconstruction errors also show coherent values with the expected deteriorations of the brain network with lowest error for CN subjects, followed by MCIs and MCIc and AD trailing behind.

*AD brain connectivity networks become less segregated*. It is clear from the different experiments that CN connectivity networks are much more segregated, with hubs around the main diagonal (intra-lobe connections). AD connectivity matrices keep approximately the same central structure but with many more connections scattered throughout the matrix. This visual impression is confirmed when we compare the small-worldness characteristic parameters of the networks. The small-world organization suggests efficient neural processing by ensuring economical communication costs. The proposed method also allowed us to visualize the strength of these connections. Again we found that AD and CN connectivity matrices share a similar structure along the diagonal (intra-lobe connections) but AD matrices also have numerous scattered weak connections (inter-lobe connections). It was also checked that the connection between the Posterior Cingulums weakens for AD patients.

*SICE can be used in clinical trials and longitudinal studies*. Differences in the connectivity matrices between the groups in clinical trials can be used to assess the efficiency of a drug. SICE has been proved to produce reliable brain connectivity models with small sample sizes. Thus, the proposed method allows lowering the sample sizes in these clinical trials. Longitudinal studies with measures of individual subjects at different points in time can be employed to construct models of the AD brain connectivity evolution. The final goal would be then to find relationships between this brain network evolution and functional deficits (He et al., 2007) so that the interconnections between them can be better understood and we could gauge the possible consequences of brain lesions and even predict the effects of possible modification (deleting or adding nodes and connections) of the network.

## 5. Conclusions

The functional consequences of network damages are a central concern in AD diagnosis. Thus, the analysis of brain networks opens avenues to understanding human brain damage and disease. SICE has proved to be an efficient tool to characterize such networks since it allows estimating conditional independence factoring out the influence of other regions. In this work we have provided a detailed analysis of the different parameters that characterize the results of this tool, and have shown how it can be used to study the main alterations in the connectome caused by AD.

## Author contributions

No significant distinction can be made between the first four co-autors; JM, AO, JG and JR have worked together and contributed equally to the reported research and writing of the paper. ADNI initiative is reponsible for the development of the database.

### Conflict of interest statement

The authors declare that the research was conducted in the absence of any commercial or financial relationships that could be construed as a potential conflict of interest.
